# Prevalence and determinants of hypertension in the adult Inuit population of Nunavik (northern Quebec, Canada)

**DOI:** 10.17269/s41997-023-00774-5

**Published:** 2023-05-08

**Authors:** Janie Allaire, Benoît Lévesque, Paul Poirier, Claudia Gagnon, Geneviève Auclair, Mélanie Lemire, Pierre Ayotte

**Affiliations:** 1 Département de médecine sociale et préventive, Université Laval, Quebec City, QC Canada; 2 Centre de recherche du CHU de Québec – Université Laval, Quebec City, QC Canada; 3 Institut national de santé publique du Québec, Quebec City, QC Canada; 4 Institut universitaire de cardiologie et de pneumologie du Québec – Université Laval, Quebec City, QC Canada; 5 Faculté de pharmacie, Université Laval, Quebec City, QC Canada; 6 Département de médecine, Université Laval, Quebec City, QC Canada; 7 Inuulitsivik Health Centre, Inukjuak, QC Canada; 8 Department of Family Medicine, McGill University, Montréal, QC Canada; 9 Institut de biologie intégrative et des systèmes, Université Laval, Quebec City, QC Canada

**Keywords:** Inuit, Hypertension, Obesity, Cardiometabolic risk factors, Socio-economic status, Inuit, hypertension, obésité, facteurs de risque cardiométabolique, statut socio-économique

## Abstract

**Objectives:**

To assess the prevalence of arterial hypertension among Inuit adults living in Nunavik (northern Quebec, Canada) in 2017 and identify its sociodemographic and lifestyle determinants.

**Methods:**

We used data obtained from 1177 Inuit adults aged ≥ 18 years who participated in the cross-sectional *Qanuilirpitaa?* Nunavik Inuit Health Survey during late summer-early fall of 2017. Resting blood pressure (BP) and anthropometric characteristics were measured during a clinical session, while sociodemographic characteristics and lifestyle habits were documented using validated questionnaires. Information on current medication was retrieved from medical files. Sex-stratified population-weighted log-binomial regressions were conducted to identify determinants of hypertension, adjusting for potential confounders.

**Results:**

Hypertension (systolic BP ≥ 140 mm Hg or diastolic BP ≥ 90 mmHg or taking antihypertensive medication) was present in 23% of the adult population and was more frequent in men than women (29% vs. 18%). About a third of hypertensive individuals (34%) were taking antihypertensive medication. These estimates are prone to biases due to the relatively low participation rate (37%). As expected, the prevalence of hypertension increased with age, but values were surprisingly elevated in 18 to 29-year-old men and women (18% and 8%, respectively) compared with 20 to 39-year-old adults of the general Canadian population (3% in both sexes, according to data from the Canadian Health Measures Survey, 2012–2015). Hypertension was associated with obesity and alcohol consumption in both men and women, and with higher socioeconomic status among men.

**Conclusion:**

This survey revealed a high prevalence of hypertension among young Nunavimmiut adults in 2017 and the need to improve hypertension diagnosis and treatment in the region. Curbing obesity and alcohol consumption, two actionable determinants of hypertension, will require improving food security and addressing the consequences of historical trauma linked to colonization.

**Supplementary Information:**

The online version contains supplementary material available at 10.17269/s41997-023-00774-5.

## Résumé

**Objectif:**

Mesurer la prévalence de l’hypertension artérielle chez les adultes vivant au Nunavik (Nord du Québec, Canada) en 2017 et identifier les caractéristiques sociodémographiques et les habitudes de vie qui lui sont associées.

**Méthodes:**

Les données ont été recueillies chez 1177 adultes ≥ 18 ans ayant participé à l’enquête de santé *Qanuilirpitaa?* auprès des Inuit du Nunavik à la fin de l’été et au début de l’automne 2017. Lors d’une visite en clinique, la tension artérielle au repos et les caractéristiques anthropométriques ont été mesurées, puis des informations concernant les caractéristiques sociodémographiques et les habitudes de vie ont été recueillies à l’aide de questionnaires validés. Une revue des dossiers médicaux a permis de documenter la prise de médicaments antihypertenseurs. Nous avons utilisé des modèles de régression log-binomiale, pondérés pour la population et ajustés pour les co-variables d’intérêt afin d’identifier les déterminants de l’hypertension chez chaque sexe.

**Résultats:**

La prévalence globale de l’hypertension était de 23 % et était plus élevée chez les hommes que chez les femmes (29 % vs. 18 %). Le tiers des hypertendus (34 %) recevait une médication antihypertensive. Ces estimés pourraient être biaisés puisque le taux de participation à l’enquête était relativement faible (37 %). Tel qu’attendu, la prévalence d’hypertension était associée à l’âge, mais des valeurs étonnamment élevées ont été notées chez les jeunes hommes et femmes âgés de 18 à 29 ans (18 % et 8 %, respectivement), comparativement aux jeunes adultes âgés de 20 à 39 ans de la population générale canadienne (3 % chez les deux sexes, selon les données de l’Enquête canadienne sur les mesures de santé, 2012–2015). Des associations avec l’obésité et la consommation d’alcool ont été notées chez les deux sexes. On a de plus observé une association avec le statut socio-économique plus élevé chez les hommes seulement.

**Conclusion:**

Cette étude a révélé une prévalence élevée d’hypertension chez les jeunes Inuit d’âge adulte résidant au Nunavik et un besoin d’améliorer le diagnostic et le traitement de la maladie dans cette région. Elle a de plus permis d’identifier deux facteurs de risque modifiables de l’hypertension dans cette population, soit l’obésité et la consommation d’alcool. Agir sur ces déterminants au Nunavik requiert l’amélioration de la sécurité alimentaire et l’atténuation des conséquences liées aux traumatismes découlant de la colonisation.

## Introduction

In recent decades, the consequences of colonization such as sedentarization and progressive shift away from traditional lifestyles and diets have led to a rise of cardiometabolic diseases among Canadian Indigenous populations (King & Furgal, 2014; Lemke & Delormier, 2017). Until recently, results from health surveys conducted in the North indicated that cardiometabolic diseases were affecting Canadian Indigenous populations unequally, with the Inuit population consistently experiencing a lower prevalence of type 2 diabetes and hypertension than First Nations and Métis (Foulds & Warburton, 2014; Leung, 2016).

The health status of Inuit living in Nunavik (Nunavimmiut) was previously documented in two major health surveys: the *Santé Québec* Survey in 1992 and the *Qanuippitaa?* Inuit Health Survey in 2004. Although Inuit’s cardiometabolic health seemed to have been less negatively impacted by the colonization-induced social transition than other Indigenous populations in Canada, an increase in the prevalence of abdominal obesity was observed between 1992 and 2004 (29.2% vs. 38.9%), primarily among women (Chateau-Degat et al., 2010a; Dewailly et al., 2007). The proportion of Nunavimmiut adults exhibiting elevated blood pressure, defined as a measured blood pressure of at least 140/90 mmHg, had doubled during the same period (6% vs. 12%; Dewailly et al., 2007). The overall prevalence of hypertension in 2004, based on blood pressure ≥ 140/90 mmHg or the use of antihypertensive medication was 19% and not different beetween sexes (Chateau-Degat et al., 2010b). Moreover, the 2004 survey revealed that more than three quarters of adults smoked daily or occasionally (Plaziac et al., 2007). The very high prevalence of smoking as well as the upward trend in obesity and hypertension noted in previous surveys warrant continued monitoring of the cardiometabolic health status in this population.

The main objective of this article is to estimate hypertension prevalence, treatment and control among Nunavimmiut adults in 2017. Second, we aimed to identify potential actionable determinants of hypertension in this population.

## Methods

### Community engagement and ethics

The *Qanuilirpitaa?* 2017 Nunavik Inuit Health Survey was set up following a resolution adopted by the Nunavik Regional Board of Health and Social Services (NRBHSS) requesting that a new health survey be conducted to update the information on the health status of Nunavimmiut. This survey was conducted in partnership with major Nunavik organizations, the Institut national de santé publique du Québec and researchers from Université Laval, McGill University and Trent University. An Inuit-led Steering Committee oversaw the preparation, conduct, data interpretation and dissemination of the survey results. A Data Management Committee (DMC) evaluated the usefulness of the research questions for the region, and approved data and biological sample requests. This committee brings together representatives from the NRBHSS and the health centres, the Kativik Regional Government, Makivik Corporation, Kativik Ilisarniliriniq, Avataq Cultural Institute and Qarjuit Youth Council. The DMC met with the researchers to discuss results and provide co-interpretation of the data that takes into consideration Inuit culture and values, according to a “two-eyed seeing approach”. Comments provided by DMC members were considered in preparing the final version of the manuscript, which was approved by the DMC. Findings were communicated to the population and partners through infographics and summaries (in Inuktitut, English and French), reports (in English) and live presentations. The *Qanuilirpitaa?* 2017 survey was also approved by the ethics committee of the Centre de recherche du CHU de Québec-Université Laval. Informed written consent was obtained from each participant and a clinical follow-up for abnormal results was undertaken when needed.

### Study population and design

In the late summer and early fall of 2017, the *Qanuilirpitaa?* Nunavik Inuit Health Survey data collection took place in the 14 communities of Nunavik. The targeted population was permanent residents of Nunavik aged ≥ 16 years. The health survey methodology was described in detail elsewhere (Hamel et al., 2020). Briefly, a stratified proportional sampling was used to select participants from the Makivik Corporation’s beneficiary list of Inuit living in Nunavik, in which age group and communities were the strata. Individuals in each stratum were selected using simple random sampling without replacement. Globally among sampled individuals, the participation rate was 36.5%. However, 79.7% of those who were successfully contacted participated in the survey. Many sampled individuals could not be contacted (because they were out of the community) or missed their appointment due to bad weather or delays in the appointment schedule.

Participants were invited on board the Canadian Coast Guard Ship (CCGS) *Amundsen* to complete questionnaires and attend clinical sessions during which biological samples and anthropometric measurements were performed. A total of 1326 individuals participated in the data collection process aboard the *Amundsen*. For the purpose of drawing comparisons with the 2004 data, analyses in the present study targeted adults (≥ 18 years old; n = 1177) and excluded pregnant women (n = 30).

### Clinical assessments, questionnaires and medical chart reviews

Resting blood pressure was measured with the ProBP 2400 Digital electronic device (Welch Allyn, NY, USA) according to the 2005 Canadian Hypertension Education Program (Hemmelgarn et al., 2005). Participants first rested comfortably for 5 min in the seated position with back support. The blood pressure cuff was placed on the supported bare arm at the heart level, as a lower position will result in an erroneously higher blood pressure. There was no talking and participants were asked not to cross their legs during the resting and measurement periods. Three measurements were taken in the same arm with the participant in the same position. The first reading was discarded and the latter two averaged. A medical chart review was performed by research nurses to obtain information on current medication of participants. The list of medication was reviewed by a physician who identified antihypertensive drugs considered when attributing the status of hypertensive to participants (Anatomical Therapeutic Classification codes: C03, C08, C09). Hypertension was defined as a systolic blood pressure ≥ 140 mm Hg or a diastolic blood pressure ≥ 90 mm Hg or the use of anti-hypertensive drugs as indicated in the medical file (Whelton et al., 2018). Treatment of hypertension was defined as a hypertensive individual who uses antihypertensive medication. Controlled hypertension was defined as a hypertensive individual who uses antihypertensive medication and has measured systolic blood pressure < 140 mm Hg and diastolic blood pressure < 90 mm Hg.

Waist circumference (WC) in centimetres (cm) was measured twice by nurses or trained interviewers and the mean of the two measurements was used (Hamel et al., 2020). A third measurement was taken if the difference between the first two values was greater than 1 cm and was used as the final value. We used sex-specific weighted WC quartiles as a measure of abdominal obesity instead of the usual cutoffs among Caucasians (≥ 88 cm in women and ≥ 102 cm in men) which may not be appropriate for different ethnic groups (Lear et al., 2010). Body mass index (BMI), calculated by dividing the weight in kilograms by the square of the height in metres (kg/m^2^), was classified according to the National Institute of Health’s scheme (National Heart, Lung & Blood Institute, 2013). A BMI value under 18.5 kg/m^2^ was classified as being underweight, ≥ 25 kg/m^2^ overweight, ≥ 30 kg/m^2^ class 1 obesity, ≥ 35 kg/m^2^ class 2 obesity and ≥ 40 kg/m^2^ class 3 obesity. The underweight and the normal BMI categories were combined because the underweight category represented < 1% of Nunavimmiut adults in 2017. Type 2 and 3 obesity categories were also combined since they represented 7% and 5% of the population in 2017, respectively.

Questionnaires were administered to participants to obtain information regarding sociodemographic characteristics and lifestyle habits. We used categorical variables for age (18–29 y; 30–49 y; ≥ 50 y), education (elementary school completed or less; secondary school not completed; secondary school completed or higher) and yearly personal income before taxes and other deductions (< $15,000; $15,000–$20,000; $20,000–$25,000; $25,000–$40,000; $40,000–$60,000; ≥ $60,000). The smoking variable comprised three categories (not at all; occasionally; daily) and the alcohol consumption variable, six categories (never; < once a month; once to 3 times a month; once to 2 times weekly; 3–6 times weekly; daily or almost daily).

### Statistical analyses

Survey weights were applied in all analyses to consider sampling methodology and non-response, allowing estimates to be inferred to the target population (Hamel et al., 2020). Bootstrap weights (500) were used to compute the variance of all estimates.

Descriptive statistics were used to provide an overview of the total population characteristics, as well as for women and men separately. Crude prevalence among women and men were compared using Chi-square tests. Sex-stratified log-binomial regression models were used to estimate prevalence ratios of hypertension according to sociodemographic characteristics (age, education, annual income), lifestyle habits (cigarette and alcohol consumption) and obesity indicators (BMI categories, WC quartiles). Models were adjusted for age (continuous) and annual income (categories), except for the age category models, which were not adjusted for any variable, and the models for education and annual income, which were adjusted for age only. Missing values for annual income (13%) were treated as a category in models. In sensitivity analyses, additional models were computed using only participants with a non-missing value for annual income.

Statistical analyses were performed with the SAS®SURVEYFREQ and SAS®GLIMMIX (with a binary distribution and a log link) procedures, version 9.4 (Cary, North Carolina, USA). Weighted estimates were obtained by including survey weights in the WEIGHT statement. Bootstrap weights were used to obtain bootstrap variances either directly from the SAS®SURVEYFREQ procedure, or by bootstrapping the SAS®GLIMMIX procedure. A bilateral significance level of 5% was used throughout.

## Results

Table 1 presents characteristics of Nunavimmiut adults aged ≥ 18 years in 2017. The mean age was 38.8 years (95% CI: 38.4 to 39.3) and about three out of four adults (73%) were < 50 years. Sixty-nine percent declared not having completed high school and almost 40% reported earning < $15,000 annually. Almost three quarters of the population declared smoking daily, whereas 6% mentioned consuming alcohol daily. A greater proportion of women reported never consuming alcohol compared to men (prevalence ratio (PR): 1.5 [95% CI: 1.1 to 2.1]).

**Table 1:** Characteristics of Nunavimmiut 18 years and over by sex, Nunavik, 2017

		Total			Women			Men	
	**N**	%	95% CI	N	%	95% CI	N	%	95% CI
Sociodemographic characteristics									
Age categories									
1–29 y	403	35.9	34.5–37.3	274	35.6	33.9–37.2	129	36.2	34.0–38.4
30–49 y	402	37.4	35.7–39.0	263	37.0	35.0–39.0	139	37.7	35.1–40.4
≥ 50 y	372	26.8	25.4–28.1	233	27.5	26.0–28.9	139	26.1	23.8–28.3
Education									
Elementary school completed or less	120	10.4	8.5–12.3	65	8.4	6.4–10.3	55	12.4	9.1–15.7
Secondary school not completed	654	58.1	55.0–61.3	431	58.7	55.2–62.3	223	57.6	52.3–62.9
Secondary school completed or higher	364	31.4	28.5–34.4	248	32.9	29.6–6.2	116	30.0	25.0–35.1
Personal annual income									
< $15,000	366	32.1	29.2–35.0	241	32.2	28.7–35.7	125	32.0	27.2–36.8
$15,000–$20,000	128	10.9	8.8–12.9	85	10.6	8.4–12.8	43	11.1	7.5–14.7
$20,000–$25,000	78	6.8	5.1–8.4	44	6.1	4.1–8.1	34	7.4	4.7–10.0
$25,000–$40,000	137	12.1	9.9–14.3	77	9.7	7.6–11.8	60	14.4	10.6–18.2
$40,000–$60,000	135	12.3	10.1–14.5	82	10.5	8.4–12.6	53	14.1	10.4–17.9
≥ $60,000	176	13.0	11.0–15.1	119	14.0	11.7–16.4	57	12.1	8.7–15.5
Missing	157	12.8	10.8–14.8	122	16.8	14.0–19.5	35	8.9	5.8- 12.0
Lifestyle habits									
Smoking									
Not at all	263	21.0	18.4–23.7	159	19.5	16.6–22.3	104	22.6	18.5–26.7
Occasionally	91	7.4	5.6–9.2	57	7.0	5.1–8.9	34	7.8	4.9–10.7
Daily	806	71.6	68.7–74.4	543	73.6	70.3–76.8	263	69.6	65.1–74.1
Alcohol consumption									
Never	186	14.2	12.1–16.4	135	17.3	14.7–19.9	51	11.4	7.9–14.8
< once a month	214	20.2	17.3–23.1	124	16.5	13.8–19.1	90	23.8	19.0–28.6
Once to 3 times a month	252	22.8	20.0–25.6	158	21.5	18.4–24.6	94	24.1	19.5–28.6
Once to 2 times a week	246	22.6	19.7–25.5	157	22.7	19.2–26.1	89	22.5	18.0–27.0
3–6 times a week	162	14.6	12.4–16.9	112	16.0	13.3–18.8	50	13.3	10.0–16.7
Daily or almost daily	55	5.5	3.9–7.1	36	6.1	4.2–8.0	19	5.0	2.5–7.5
Obesity indicators									
Body mass index									
Underweight + Normal	469	42.1	39.0–45.3	275	35.3	31.9–38.7	194	48.9	43.5–54.3
Overweight	311	25.7	22.9–28.5	206	27.4	24.3–30.6	105	24.1	19.6–28.5
Type 1 obesity	242	20.0	17.5–22.4	171	22.0	19.2–24.9	71	17.9	14.0–21.9
Type 2 + 3 obesity	153	12.2	10.0–14.3	116	15.2	12.6–17.9	37	9.2	6.0–12.4
Waist circumference (cm; median, Q1-Q3)									
		92.4	80.1–105.5		94.6	83.1–105.8		89.2	79.6–104.0

Results are presented as unweighted N and weighted proportions (%) with their associated 95% confidence interval, unless stated otherwise

*CI* confidence interval; *Q1* first quartile; *Q3* third quartile

The underweight or normal category was defined as a BMI < 25 kg/m^2^, the overweight as a BMI ≥ 25 but < 30 kg/m^2^, type 1 obesity class was defined as a BMI between 30 and 34 kg/m^2^, and type 2 + 3 categories were defined as a BMI ≥ 35 kg/m^2^

The overall prevalence of obesity was 32% in this population (BMI ≥ 30 kg/m^2^). The prevalence of type 2 and 3 obesity was higher among women than among men (PR: 1.7 [1.1 to 2.5]), whereas men had a higher prevalence of underweight/normal BMI than women (PR: 1.4 [1.2 to 1.6]). The overall median WC was 92.4 cm, the median value in women exceeding that of men by 5.4 cm (94.6 cm [93.0 to 96.1] vs. 89.2 cm [86.8 to 91.7], respectively).

The global prevalence of hypertension among Nunavimmiut was 23%: 19% displayed elevated blood pressure during the clinical session, whereas 8% had use of antihypertensive medication mentioned in their medical file (Table 2). About a third of hypertensive individuals (34%) were being treated for their condition and effective control was noted in about half (52%) of those treated.

**Table 2:** Hypertension prevalence, treatment and control, by sex and age group, among Nunavimmiut 18 years and over, 2017

	Prevalence						Treatment		Control		
	Total		BP ≥ 140/90 mm Hg		Antihypertensive medication						
	%	95%CI	%	95% CI	%	95% CI	%	95% CI	%		95% CI
Both sexes	23.2	20.6–25.9	19.2	16.6–21.8	7.8	6.2–9.4	33.5	27.3–39.6	17.4		12.6–22.4
18–29 y	12.9	8.7–17.2	12.4	8.2–16.6	NP	–	NP	–	NP		–
30–49 y	19.6	15.3–24.0	16.7	12.6–20.8	4.2	1.8–6.5	21.1	10.3–32.0	14.9		4.9–25.0
≥ 50 y	42.1	37.0–47.3	31.9	26.9–36.9	21.2	17.0–25.3	50.2	41.3–59.2	24.3		17.8–30.9
Women	17.5	14.7–20.2	13.6	11.0–16.2	5.7	4.3–7.0	32.5	24.8–40.1	21.9		15.2–28.5
18–29 y	7.5	3.9–11.1	7.5	3.9–11.1	0	–	0	–	0		–
30–49 y	15.4	11.0–19.8	13.8	9.5–18.1	2.4	0.9–3.9	15.5	6.0–24.9	10.6		1.8–19.4
≥ 50 y	33.1	26.6–39.7	21.4	15.4–27.4	17.4	12.9–22.0	52.6	40.9–64.3	35.3		24.5–46.1
Men	28.9	24.2–33.6	24.7	20.2–29.1	9.8	6.9–12.8	34.0	25.4–42.7	14.8		7.9–21.6
18–29 y	18.2	10.4–26.0	17.1	9.5–24.6	NP	–	NP	–	NP		–
30–49 y	23.7	16.4–31.0	19.5	13.0–26.1	5.9	1.4–10.3	24.7	8.0–41.4	17.7	2.2–33.2	
≥ 50 y	51.5	42.6–60.3	42.7	34.0–51.4	25.0	18.1–31.9	48.6	36.2–61.1	17.0	8.6–25.5	

Results are presented as weighted proportions (%) with their associated 95% confidence interval

*NP* not presented (n < 5)

*BP* blood pressure; *CI* confidence interval

Hypertension was defined as a systolic BP ≥ 140 mm Hg or a diastolic BP ≥ 90 mm Hg or the use of antihypertensive medication. Treatment (of hypertension) was defined as a hypertensive individual who uses antihypertensive medication. Controlled (hypertension) was defined as a hypertensive individual who uses antihypertensive medication and has measured systolic BP < 140 mm Hg and diastolic BP < 90 mm Hg

Hypertension was more frequent among men than among women (29% vs 18%; PR: 1.7 [1.3 to 2.1]). Among women, the prevalence of hypertension doubled from 8% in the younger age group to 15% in the middle-age group, and doubled again to reach 33% in the older age group (Table 3). The prevalence of hypertension in young men was 18%, 24% in middle-aged men and more than doubled to reach 52% in older men (Table 4).

**Table 3:** Prevalence ratio (PR) of hypertension according to sociodemographic characteristics and lifestyle determinants among women aged 18 years and over, Nunavik, 2017

	Prevalence	95% CI	PR	95% CI
Sociodemographic characteristics				
Age category, years				
18–29 (ref.)	7.5	4.5–12.5	-	-
30–49	15.4	11.5–20.7	**2.06**	**1.14–3.73**
50 +	33.1	27.2–40.3	**4.43**	**2.54–7.73**
Education				
Elementary school completed or less (ref.)	12.7	7.8–20.6	-	-
Secondary school not completed	13.5	10.5–17.4	1.07	0.66–1.74
Secondary school completed or higher	15.8	11.7–21.2	1.24	0.75–2.07
Personal annual income				
< $15,000 (ref.)	10.4	7.0–15.5	-	-
$15,000–$20,000	18.3	11.5–29.3	**1.77**	**1.00–3.12**
$20,000–$25,000	18.6	10.1–34.3	1.79	0.88–3.66
$25,000–$40,000	14.1	8.6–23.1	1.36	0.73–2.54
$40,000–$60,000	17.6	11.4–27.1	1.70	0.96–2.99
≥ $60,000	12.6	8.4–19.0	1.22	0.70–2.13
Missing	18.4	12.7–26.7	**1.78**	**1.08–2.92**
Lifestyle habits				
Smoking				
Not at all (ref.)	16.5	11.2–24.1	-	-
Occasionally	18.9	10.9–32.6	1.15	0.64–2.05
Daily	12.9	10.0–16.6	0.78	0.53–1.15
Alcohol consumption				
Never (ref.)	9.9	5.9–16.6	-	-
Less than once a month	12.8	7.9–20.9	1.30	0.72–2.35
Once to 3 times a month	13.0	8.0–21.1	1.32	0.71–2.46
Once to 2 times a week	12.4	7.8–19.8	1.26	0.66–2.39
3 times a week or more	20.7	14.6–29.3	**2.09**	**1.19–3.69**
Obesity indicators				
Body mass index				
Underweight + Normal (ref.)	9.1	6.1–13.6	-	-
Overweight	13.7	9.8–19.2	1.51	0.94–2.41
Type 1 obesity	11.6	7.9–17.1	1.28	0.78–2.10
Type 2 + 3 obesity	29.8	21.9–40.5	**3.27**	**2.07–5.17**
Waist circumference				
Q1 (63.0–82.9 cm) (ref.)	9.7	6.0–15.8	-	-
Q2 (83.0–94.5 cm)	11.8	7.8–17.7	1.21	0.67–2.20
Q3 (94.6–105.7 cm)	12.4	8.5–18.2	1.28	0.72–2.27
Q4 (105.8–142.0 cm)	22.4	16.7–30.0	**2.30**	**1.41–3.75**

Results are presented as weighted prevalence ratios with their associated 95% confidence interval

*CI* confidence interval; *ref*. reference category

Models were adjusted for age (continuous) and personal annual income categories, except for the model for age categories which was not adjusted for any variable and the models for the education and personal annual income which were adjusted for age only

Bold indicates *p* < 0.05

**Table 4:** Prevalence ratio (PR) of hypertension according to sociodemographic characteristics and lifestyle determinants among men aged 18 years and over, Nunavik, 2017

	Prevalence	95% CI	PR	95% CI
Sociodemographic characteristics				
Age category, years				
18–29 (ref.)	18.2	11.5–28.7	-	-
30–49	23.7	17.4–32.3	1.30	0.76–2.22
50 +	51.5	43.3–61.2	**2.83**	**1.75–4.57**
Education				
Elementary school completed or less (ref.)	21.9	13.9–34.6	-	-
Secondary school not completed	25.6	19.9–33.0	1.17	0.70–1.95
Secondary school completed or higher	34.1	25.3–45.9	**1.55**	**1.00–2.42**
Personal annual income				
< $15,000 (ref.)	24.0	17.3–33.2	-	-
$15,000–$20,000	26.5	15.0–46.7	1.11	0.58–2.10
$20,000–$25,000	21.9	10.4–46.0	0.92	0.41–2.03
$25,000–$40,000	23.6	15.0–37.3	0.99	0.58–1.69
$40,000–$60,000	26.5	15.7–44.7	1.11	0.61–1.99
≥ $60,000	39.9	30.3–52.7	**1.67**	**1.12–2.47**
Missing	38.5	25.0–59.1	1.61	0.98–2.63
Lifestyle				
Smoking				
Not at all (ref.)	26.2	18.6–36.9	-	-
Occasionally	45.2	30.1–67.9	**1.72**	**1.08–2.74**
Daily	25.6	20.0–32.7	0.97	0.68–1.39
Alcohol consumption				
Never (ref.)	23.9	15.3–37.5	-	-
Less than once a month	21.1	14.1–31.7	0.88	0.50–1.55
Once to 3 times a month	23.6	15.0–37.2	0.99	0.55–1.78
Once to 2 times a week	26.6	18.8–37.7	1.11	0.67–1.85
3 times a week or more	47.0	34.9–63.3	**1.96**	**1.26–3.06**
Obesity indicators				
Body mass index				
Underweight + Normal (ref.)	11.7	7.4–18.7	-	-
Overweight	37.2	26.9–51.4	**3.17**	**1.74–5.77**
Type 1 obesity	40.1	28.3–56.8	**3.42**	**1.87–6.25**
Type 2 + 3 obesity	57.7	40.1–83.1	**4.92**	**2.60–9.30**
Waist circumference				
Q1 (68.0–79.5 cm) (ref.)	8.1	3.3–19.6	-	-
Q2 (79.6–89.2 cm)	13.4	7.8–22.9	1.66	0.61–4.56
Q3 (89.3–103.9 cm)	38.3	28.0–52.3	**4.74**	**1.84–12.22**
Q4 (104.0–143.0 cm)	48.3	35.8–65.2	**5.99**	**2.27–15.80**

Results are presented as weighted prevalence ratios with their associated 95% confidence interval

*CI* confidence interval; *ref.* reference category

Models were adjusted for age (continuous) and personal annual income categories, except for the model for age categories which was not adjusted for any variable and the models for the education and personal annual income which were adjusted for age only

Bold indicates *p* < 0.05

While education and socio-economic status were not consistently associated with hypertension among women (Table 3), men who completed secondary school or higher were 55% more at risk of hypertension than those who completed elementary school or less (Table 4). Men earning > $60,000 annually also displayed a 67% greater prevalence of hypertension than their counterparts earning < $15,000.

Drinking ≥ 3 times weekly approximately doubled the risk of hypertension compared to abstainers among both women and men. Smoking was not associated with hypertension in women, and in men, only those who smoked occasionally (less than daily smoking) exhibited a greater prevalence of hypertension compared to non-smokers (72% higher).

Obesity indicators (BMI and WC) were associated with hypertension in both sexes. In men, graded relationships were noted between BMI categories or WC quartiles and the prevalence of hypertension. Overweight men (BMI of 25 to 29.9 kg/m^2^) displayed a threefold greater risk of hypertension compared to their lean counterparts (BMI < 25 kg/m^2^), and prevalence values were five to sixfold higher in the highest obesity categories (defined by a BMI ≥ 35 kg/m^2^ or a WC value in the upper quartile) compared to the lowest. In contrast to men, women displayed an increased prevalence of hypertension only in the highest obesity class (PR = 3.3, BMI ≥ 35 vs < 25 kg/m^2^; PR = 2.3, WC Q4 vs Q1).

In sensitivity analyses, models were computed using only participants with a non-missing value for annual income and similar results were obtained (see Tables S1 and S2, supplementary material).

## Discussion

Data from the *Qanuilirpitaa?* survey indicate that in 2017, hypertension was present in close to one out of four Nunavimmiut aged ≥ 18 years, disproportionately affecting men compared to women. Age, alcohol consumption and obesity were associated with a higher prevalence of hypertension in both men and women. Public health actions that aim at reducing alcohol consumption and maintaining a healthy body weight may help in curbing the rise of hypertension in the Nunavik population.

The age-adjusted prevalence of hypertension in 2017 (23%) was up 6 percentage points from that of 17% documented in the framework of the previous Nunavik Inuit Health Survey conducted in 2004 (Allaire et al., 2021a). The increase was most striking among men, for whom the prevalence increased from 19% in 2004 to 28% in 2017, compared to women whose prevalence only marginally increased from 14% to 17% during the same period (Allaire et al., 2021a; Dewailly et al., 2007). Results of surveys conducted over the past decades in Nunavik and Greenland also indicate a greater prevalence of hypertension in men than in women (Bjerregaard et al., 2002, 2003; Riva et al., 2016).

As observed in all populations surveyed throughout the world (Mills et al., 2016), the prevalence of hypertension increased with age in the Nunavik population. Especially noteworthy is the elevated prevalence of hypertension documented in younger age Nunavimmiut (18 to 29 years). Comparisons with prevalence data for Canadians aged 20 to 39 years from the general population (DeGuire et al., 2019), obtained in the framework of the Canadian Health Measures Survey (combined cycles 3 and 4, 2012–2015), reveal a more than twofold greater prevalence of hypertension among young Nunavimmiut women (8% vs. 3%) and a sixfold greater prevalence among young Nunavimmiut men (18% vs. 3%). Care should be taken when comparing hypertension prevalence between these surveys because of methodological differences. Most notably, six blood pressure measurements were conducted in the Canadian survey, compared to three in the present study (the first measurement was discarded in both surveys). The greater number of readings used may lead to lower blood pressures and hypertension prevalence (Bryan et al., 2010). Nevertheless, values noted in young Nunavimmiut are also greater than those of similarly aged individuals living in high-income countries and are similar to those in low-to-middle income countries (Mills et al., 2016). Hypertension in young adults is of great concern and should be prevented as it may lead to cardiovascular events in later life (Hamrahian and Falkner, 2022).

In order to manage and control blood pressure among Nunavimmiut, improvement in diagnosis and treatment of hypertension will be required. Indeed, results from the present study indicate that 34% of hypertensive individuals were taking antihypertensive medication, according to information extracted from their medical file. In comparison, 82% of hypertensive Canadians aged 20 to 79 years reported taking antihypertensive medication during the last month, according to data from the Canadian Health Measures Survey in 2014–2015 (DeGuire et al., 2019). While the definition of hypertension treatment is somewhat different between the two studies, the large gap in the prevalence of treatment between the Nunavik population and the general Canadian population points to the need to improve diagnosis of hypertension among Nunavimmiut. Hypertension control in the present study (17%) was also lower than in the Canadian population (66%) (DeGuire et al., 2019).

Obesity is a well-known risk factor of hypertension, although some obese people do not develop hypertension or other characteristics of the metabolically unhealthy phenotype (Abiri et al., 2022). Our results indicate that while obesity affects a greater proportion of women than of men, it has a greater impact on the prevalence of hypertension among men. Indeed, nearly half of men in the highest WC quartile had hypertension, compared to one out of four women in the highest WC quartile (Tables 3 and 4). Hence, metabolically healthy obesity may be more prevalent among women than among men in this population (Powell-Wiley et al., 2021). A more complete assessment of metabolic outcomes should be conducted to define sex-specific WC cutoffs for metabolically-dangerous abdominal obesity in this population.

A separate study focusing on dietary profiles revealed that many Nunavimmiut are high consumers of refined and processed foods that have been associated with a greater likelihood of displaying an unhealthy metabolic phenotype (Aker et al., this issue). Discussions held during co-interpretation sessions further revealed that Nunavimmiut frequently add seasoning high in sodium or soy sauce while consuming country foods. Sodium intake is a well-known determinant of blood pressure (Sacks et al., 2001) that may contribute to the risk of hypertension in Nunavimmiut. Among normotensive individuals who participated in the 2004 *Qanuippitaa?* survey, daily sodium intake assessed by a 24-h dietary recall was found to be positively associated with the systolic blood pressure of Nunavimmiut adults, after adjustment for confounders (Chateau-Degat et al., 2012). In the latter survey, both daily sodium intake and systolic blood pressure were significantly higher in men than in women. Unfortunately, sodium intake could not be measured in the *Qanuilirpitaa*? survey and therefore its influence on hypertension could not be evaluated in the present study. Further analyses will be required to better identify dietary habits favouring hypertension in the Nunavik population.

Data from the present survey indicated that higher education level and personal annual income were positively associated with the prevalence of hypertension, especially in men. An inverse U-shape association was previously observed between socioeconomic conditions and blood pressure among Inuit in Greenland (Riva et al., 2016). This association may reflect a protective role of traditional lifestyle in more remote and traditional villages and a better access to health resources and services among wealthier villages (Riva et al., 2016). Psychosocial factors such as mastery, control and social support are also likely to be influenced positively by traditional lifestyle and culture and may mediate the association between socio-environmental risk factors and the risk of cardiometabolic diseases (Daniel et al., 2011). On the other hand, social transition may act as a chronic stressor, induce physiological stress response and consequently exert a negative impact on cardiometabolic health (Daniel et al., 2011).

In the present study, Nunavimmiut men who reported smoking occasionally displayed a higher prevalence of hypertension than non-smokers, but the prevalence was similar among daily smokers and non-smokers. Because of the transversal nature of the study, interpretation of these results is not straightforward. They may reflect a reduction in cigarette consumption—from daily to occasional—among smokers who had received a diagnosis of high blood pressure. Previous data on the relationship between blood pressure and smoking among populations living in the Arctic have yielded inconsistent results. Mean blood pressure was higher in both non-smokers and former smokers compared to current smokers in Greenland Inuit (Bjerregaard et al., 2002). Being a non-smoker was also associated with increased blood pressure in a joint analysis of data from surveys conducted in Inuit from Alaska, Canada, and Greenland (Bjerregaard et al., 2003). However, the smoking status was no longer associated with hypertension in Canadian Inuit (Kivalliq and Nunavik) when analyzed separately (Bjerregaard et al., 2003). Multiple contextual factors (e.g., housing conditions, psychosocial stressors, food insecurity, obesity phenotype) might influence the association between smoking and blood pressure among Nunavimmiut (Bougie & Kohen, 2018). Smoking may also be a way to cope with stress or distress and determinants of smoking appear to be gender-specific (Bougie & Kohen, 2018). Several studies conducted elsewhere in the world have identified smoking as a risk factor for hypertension (Bowman et al., 2007; Ezzati et al., 2002; Gao et al., 2017; Jatoi et al., 2007), although some studies documented opposite findings (Okubo et al., 2002, 2004).

Higher alcohol consumption frequency was associated with a greater prevalence of hypertension in both women and men in 2017. In 2004, alcohol consumption (yes vs. no) was also associated with the prevalence of hypertension in Nunavimmiut (Chateau-Degat et al., 2010b), but alcohol consumption was not related to blood pressure in Inuit living in Greenland and Inuit migrants in Denmark between 1998 and 2001 (Bjerregaard et al., 2002). The relationship between alcohol consumption and hypertension is well established, but the mechanisms are not completely understood (Husain et al., 2014). Similarly to smoking, alcohol may be a way to cope with stress or distress (Seale et al., 2006).

### Limitations of the study

Results presented here must be interpreted in the context of some limitations. The low participation rate among sampled individuals (36.5%) might have introduced a selection bias that could not be fully corrected with weighting, but it could be partially offset by the high participation rate among reached individuals (80%). Discussions of survey results during co-interpretation sessions indicated that the *Qanuilirpitaa?* 2017 survey had succeeded in capturing the diversity of socio-economic status, food security status, dietary habits, and Inuit culture adherence encountered in the Nunavik population (data available in following thematic reports: Allaire et al., 2021b; Furgal et al., 2022; Muckle et al., 2020; Riva et al., 2020). This provides further confidence in the representativeness of our population sample.

Blood pressure was measured on a single occasion during the visit on board the *Amundsen*. Ambulatory monitoring would have provided a more accurate assessment of the blood pressure status of participants. Also, antihypertensive medication may be prescribed for conditions other than hypertension. However, most individuals were classified as hypertensive based on high blood pressure measurements during their visit (83%). Therefore, misclassification based on the use of medication for other conditions is unlikely to have resulted in a significant overestimation of the prevalence of hypertension.

Using a cross-sectional design, it is assumed that exposure to determinants at the time of the survey represents the past exposure, which may not always be the case. This assumption has an important impact on the interpretation of the results since high blood pressure develops progressively over time. However, our objective was not to infer causality but to identify determinants of hypertension amenable to public health measures in Nunavik. Some determinants such as daily smoking and weekly alcohol consumption or more were highly prevalent among Nunavimmiut (≈ 70%). The low variability noted for these variables may cause instability in the corresponding estimates. However, the narrow confidence intervals obtained in determinants analyses suggest that estimates presented here are relatively precise and accurate.

## Conclusion

Data from the *Qanuilirpitaa?* 2017 survey indicate that hypertension has become a public health concern among Nunavimmiut, especially among men. Obesity and alcohol consumption were identified as determinants of high blood pressure in both sexes, but the impact of obesity on the risk of hypertension is much stronger among men. Reducing alcohol consumption and curbing obesity are actionable risk factors of hypertension in Nunavik. Acting on these proximal determinants will require addressing upstream causal factors—historical trauma and food insecurity—that are affecting health and well-being of Nunavimmiut.

## Contributions to knowledge

What does this study add to existing knowledge?

- Hypertension is relatively frequent in Nunavik, especially among young adults, and even more so among young men.
- Hypertension treatment is less frequent in Nunavik than in the general Canadian population.
- Obesity and elevated alcohol consumption are determinants of hypertension that warrant more research in the Inuit context. Obesity has a much bigger impact on hypertension in men than in women.

What are the key implications for public health interventions, practice, or policy?

- The reinforcement of programs to prevent excessive alcohol consumption may help prevent hypertension, among many other benefits. Improving life conditions that influence alcohol consumption is required.
- Findings can be used in advocacy efforts toward structural policy actions to reduce obesity through better access to healthy nutritious foods.
- Increasing the access to primary care for hypertension might be considered among other healthcare needs.

### Supplementary Information

Below is the link to the electronic supplementary material.

Supplementary file1 (DOCX 51 kb)

## Data Availability

The survey data are owned by Inuit and can be accessed through a request made to *Qanuilirpitaa?* 2017 DMC (nunavikhealthsurvey@ssss.gouv.qc.ca).
