# Supplementary material for: Prevalence and determinants of hypertension in the adult Inuit population of Nunavik (northern Quebec, Canada)
Source: Can J Public Health. 2023 May 8;115(Suppl 1):168–79. doi: 10.17269/s41997-023-00774-5 (PMC10830977; doi:10.17269/s41997-023-00774-5)
Supplement: Supplementary file 1 — Supplementary file1 (DOCX 51 kb) [file 41997_2023_774_MOESM1_ESM.docx]

**SUPPLEMENTAL MATERIAL**

**Prevalence and determinants of hypertension in the adult Inuit population
 of Nunavik (northern Quebec, Canada)**

Janie Allaire^1,2,3^, Benoît Lévesque^1,3^, Paul Poirier^4,5^, Claudia Gagnon^2,4,6^, Geneviève Auclair^7,8^ Mélanie Lemire^1,2,9^, Pierre Ayotte^1,2,3^

^1^ Département de médecine sociale et préventive, Université Laval, Québec, Canada

^2^ Centre de recherche du CHU de Québec – Université Laval, Québec, Canada

^3^ Institut national de santé publique du Québec, Québec, Canada.

^4^ Institut universitaire de cardiologie et de pneumologie du Québec – Université Laval, Québec, Canada

^5^ Faculté de pharmacie, Université Laval, Québec, Canada

^6^ Département de médecine, Université Laval, Québec, Canada

^7^ Inuulitsivik Health Center, Inukjuak, Québec, Canada

^8^ Department of Family Medicine, McGill University, Montréal, Québec, Canada

^9^ Institut de biologie intégrative et de systèmes, Université Laval, Québec, Québec, Canada

**Corresponding author**: Pierre Ayotte
Université Laval and Institut national de santé publique du Québec

945, avenue Wolfe
Québec, QC, Canada G1V 5B3

Tel: 418-650-5115; Email: pierre.ayotte@inspq.qc.ca

| **Table S1. Prevalence ratio (PR) of hypertension according to sociodemographic characteristics and lifestyle determinants among women aged 18 years and over, Nunavik, 2017 — Subsample of 648 women with non-missing value for personal annual income** | | | | | |
| --- | --- | --- | --- | --- | --- |
|  | **Prevalence** | **95% CI** | **PR** | **95% CI** |  |
| **Sociodemographic characteristics** |  |  |  |  |  |
| Age category, years |  |  |  |  |  |
| *18 - 29 (ref.)* | 5.8 | (3.1 - 11.0) | - | - |  |
| *30 – 49* | 14.3 | (10.4 - 19.6) | **2.45** | **(1.21 - 4.97)** |  |
| *50 +* | 31.1 | (24.7 - 39.3) | **5.34** | **(2.68 - 10.61)** |  |
| Education |  |  |  |  |  |
| *Elementary school completed or less (ref.)* | 13.5 | (8.1 - 22.6) | - | - |  |
| *Secondary school not completed* | 12.4 | (9.2 - 16.6) | 0.91 | (0.55 - 1.53) |  |
| *Secondary school completed or higher* | 13.3 | (9.6 - 18.5) | 0.98 | (0.56 - 1.74) |  |
| Personal annual income |  |  |  |  |  |
| *< $15 000 (ref.)* | 9.9 | (6.6 - 14.9) | - | - |  |
| *$15 000 > $20 000* | 16.9 | (10.6 - 26.8) | **1.71** | **(1.00 - 2.91)** |  |
| *$20 000 > $25 000* | 17.4 | (9.6 - 31.7) | 1.76 | (0.88 - 3.52) |  |
| *$25 000 > $40 000* | 13.4 | (8.2 - 22.0) | 1.36 | (0.74 - 2.50) |  |
| *$40 000 > $60 000* | 16.9 | (11.0 - 26.0) | 1.71 | (0.98 - 2.97) |  |
| *$60 000 >* | 11.9 | (7.9 - 18.0) | 1.21 | (0.70 - 2.09) |  |
| **Lifestyle habits** |  |  |  |  |  |
| Smoking |  |  |  |  |  |
| *Not at all (ref.)* | 16.1 | (10.8 - 23.9) | - | - |  |
| *Occasionally* | 13.0 | (5.8 - 29.0) | 0.81 | (0.35 - 1.85) |  |
| *Daily* | 11.6 | (8.8 - 15.4) | 0.72 | (0.48 - 1.09) |  |
| Alcohol consumption |  |  |  |  |  |
| *Never (ref.)* | 8.9 | (5.1 - 15.6) | - | - |  |
| *Less than once a month* | 11.1 | (6.4 - 19.2) | 1.24 | (0.63 - 2.45) |  |
| *Once to 3 times a month* | 10.7 | (6.1 - 18.6) | 1.20 | (0.58 - 2.49) |  |
| *Once to 2 times a week* | 10.8 | (6.4 - 18.2) | 1.21 | (0.58 - 2.54) |  |
| *3 times a week or more* | 19.8 | (13.3 - 29.5) | **2.22** | **(1.23 - 4.02)** |  |
| **Obesity indicators** |  |  |  |  |  |
| Body mass index |  |  |  |  |  |
| *Underweight + Normal (ref.)* | 8.3 | (5.4 - 12.7) | - | - |  |
| *Overweight* | 10.3 | (7.0 - 15.3) | 1.24 | (0.74 - 2.08) |  |
| *Type 1 obesity* | 11.2 | (7.5 - 16.7) | 1.35 | (0.83 - 2.20) |  |
| *Type 2 + 3 obesity* | 28.3 | (19.8 - 40.5) | **3.40** | **(2.06 - 5.62)** |  |
| Waist circumference |  |  |  |  |  |
| *Q1 (63.0 – 82.9 cm) (ref.)* | 10.5 | (6.5 - 17.0) | - | - |  |
| *Q2 (83.0 – 94.5 cm)* | 8.0 | (4.8 - 13.1) | 0.76 | (0.39 - 1.45) |  |
| *Q3 (94,6 – 105.7 cm)* | 11.6 | (7.7 - 17.6) | 1.11 | (0.63 - 1.95) |  |
| *Q4 (105.8 – 142.0 cm)* | 20.3 | (14.6 - 28.4) | **1.94** | **(1.20 - 3.13)** |  |

Estimates are expressed as prevalence ratios and associated 95% confidence interval.

CI: confidence interval; ref.: category of reference.

Models were adjusted for age (continuous) and personal annual income categories, except for the model for age categories which was not adjusted for any variable and the models for the education and personal annual income which were adjusted for age only.

Bold indicates P<0.05.

| **Table S2. Prevalence ratio (PR) of hypertension according to sociodemographic characteristics and lifestyle determinants among men aged 18 years and over, Nunavik, 2017 — Subsample of 372 men with non-missing value for personal annual income** | | | | | |
| --- | --- | --- | --- | --- | --- |
|  | **Prevalence** | **95% CI** | **PR** | **95% CI** |  |
| **Sociodemographic characteristics** |  |  |  |  |  |
| Age category, years |  |  |  |  |  |
| *18 - 29 (ref.)* | 19.1 | (11.9 - 30.7) | - | - |  |
| *30 - 49* | 19.9 | (14.5 - 27.2) | 1.04 | (0.60 - 1.82) |  |
| *50 +* | 51.8 | (43.0 - 62.4) | **2.71** | **(1.64 - 4.47)** |  |
| Education |  |  |  |  |  |
| *Elementary school completed or less (ref.)* | 23.2 | (14.5 - 37.1) | - | - |  |
| *Secondary school not completed* | 23.8 | (18.2 - 31.1) | 1.03 | (0.60 - 1.74) |  |
| *Secondary school completed or higher* | 32.6 | (23.8 - 44.6) | 1.40 | (0.89 - 2.21) |  |
| Personal annual income |  |  |  |  |  |
| *< $15 000 (ref.)* | 23.9 | (17.2 - 33.2) | - | - |  |
| *$15 000 > $20 000* | 26.5 | (15.1 - 46.6) | 1.11 | (0.59 - 2.10) |  |
| *$20 000 > $25 000* | 21.8 | (10.4 - 45.5) | 0.91 | (0.41 - 2.01) |  |
| *$25 000 > $40 000* | 23.6 | (15.0 - 37.2) | 0.99 | (0.58 - 1.68) |  |
| *$40 000 > $60 000* | 26.4 | (15.6 - 44.7) | 1.10 | (0.62 - 1.98) |  |
| *$60 000 >* | 39.6 | (29.9 - 52.4) | **1.66** | **(1.12 - 2.45)** |  |
| **Lifestyle** |  |  |  |  |  |
| Smoking |  |  |  |  |  |
| *Not at all (ref.)* | 23.9 | (16.4 - 34.9) | - | - |  |
| *Occasionally* | 43.2 | (28.8 - 64.7) | **1.81** | **(1.12 - 2.92)** |  |
| *Daily* | 25.2 | (19.6 - 32.3) | 1.05 | (0.72 - 1.55) |  |
| Alcohol consumption |  |  |  |  |  |
| *Never (ref.)* | 24.3 | (15.4 - 38.4) | - | - |  |
| *Less than once a month* | 17.7 | (11.1 - 28.3) | 0.73 | (0.39 - 1.37) |  |
| *Once to 3 times a month* | 23.4 | (14.9 - 36.8) | 0.96 | (0.53 - 1.73) |  |
| *Once to 2 times a week* | 25.7 | (17.4 - 38.0) | 1.06 | (0.61 - 1.85) |  |
| *3 times a week or more* | 46.4 | (34.0 - 63.3) | **1.91** | **(1.22 - 3.00)** |  |
| **Obesity indicators** |  |  |  |  |  |
| Body mass index |  |  |  |  |  |
| *Underweight + Normal (ref)* | 12.5 | (7.8 - 20.0) | - | - |  |
| *Overweight* | 36.4 | (25.9 - 51.2) | **2.91** | **(1.57 - 5.37)** |  |
| *Type 1 obesity* | 35.2 | (23.7 - 52.3) | **2.81** | **(1.47 - 5.35)** |  |
| *Type 2 + 3 obesity* | 55.7 | (37.4 - 83.0) | **4.45** | **(2.29 - 8.65)** |  |
| Waist circumference |  |  |  |  |  |
| *Q1 (68.0 – 79.5cm) (ref.)* | 9.0 | (3.7 - 21.7) | - | - |  |
| *Q2 (79.6 – 89.2 cm)* | 13.6 | (8.0 - 23.3) | 1.52 | (0.56 - 4.14) |  |
| *Q3 (89.3 – 103.9 cm)* | 37.5 | (26.8 - 52.6) | **4.19** | **(1.60 - 10.94)** |  |
| *Q4 (104.0 – 143.0 cm)* | 45.8 | (32.8 - 63.7) | **5.10** | **(1.92 - 13.60)** |  |

Estimates are expressed as prevalence ratios and associated 95% confidence interval.

CI: confidence interval; ref.: category of reference.

Models were adjusted for age (continuous) and personal annual income categories, except for the model for age categories which was not adjusted for any variable and the models for the education and personal annual income which were adjusted for age only.

Bold indicates P<0.05.
